# Elucidating the Structural Basis of the Intracellular pH Sensing Mechanism of TASK-2 K_2_P Channels

**DOI:** 10.3390/ijms21020532

**Published:** 2020-01-14

**Authors:** Daniel Bustos, Mauricio Bedoya, David Ramírez, Guierdy Concha, Leandro Zúñiga, Niels Decher, Erix W. Hernández-Rodríguez, Francisco V. Sepúlveda, Leandro Martínez, Wendy González

**Affiliations:** 1 Centro de Bioinformática y Simulación Molecular, Universidad de Talca, Talca 3460000, Chile; dbustos@ucm.cl (D.B.); maurobedoyat@gmail.com (M.B.); 2 Departamento de Computación e Industrias, Facultad de Ciencias de la Ingeniería, Universidad Católica del Maule, Talca 3460000, Chile; 3 Instituto de Ciencias Biomédicas, Universidad Autónoma de Chile, Santiago 8380453, Chile; david.ramirez@uautonoma.cl; 4 Centro de Investigaciones Médicas, Escuela de Medicina, Universidad de Talca, Talca 3460000, Chile; guierdy@gmail.com (G.C.); lzuniga@utalca.cl (L.Z.); 5 Magíster en Gestión de Operaciones, Facultad de Ingeniería (Campus Los Niches), Universidad de Talca, Talca 3460000, Chile; 6 Programa de Investigación Asociativa en Cáncer Gástrico (PIA-CG), Universidad de Talca, Talca 3460000, Chile; 7 Institute for Physiology and Pathophysiology, Vegetative Physiology, University of Marburg, D-35037 Marburg, Germany; decher@staff.uni-marburg.de; 8 Escuela de Química y Farmacia, Facultad de Medicina, Universidad Católica del Maule, Talca 3460000, Chile; ehernandez@ucm.cl; 9 Centro de Estudios Científicos (CECs), Avenida Arturo Prat 514, Valdivia 5110466, Chile; 10 Institute of Chemistry and Center for Computing in Engineering & Science, University of Campinas, Campinas 13083-861 SP, Brazil; 11 Millennium Nucleus of Ion Channels-Associated Diseases (MiNICAD), Universidad de Talca, Talca 3460000, Chile

**Keywords:** K_2_P channels, TASK-2 channels, structure–function relationships, homology modeling, modeling of protein dynamics

## Abstract

Two-pore domain potassium (K_2_P) channels maintain the cell’s background conductance by stabilizing the resting membrane potential. They assemble as dimers possessing four transmembrane helices in each subunit. K_2_P channels were crystallized in “up” and “down” states. The movements of the pore-lining transmembrane TM4 helix produce the aperture or closure of side fenestrations that connect the lipid membrane with the central cavity. When the TM4 helix is in the up-state, the fenestrations are closed, while they are open in the down-state. It is thought that the fenestration states are related to the activity of K_2_P channels and the opening of the channels preferentially occurs from the up-state. TASK-2, a member of the TALK subfamily of K_2_P channels, is opened by intracellular alkalization leading the deprotonation of the K245 residue at the end of the TM4 helix. This charge neutralization of K245 could be sensitive or coupled to the fenestration state. Here, we describe the relationship between the states of the intramembrane fenestrations and K245 residue in TASK-2 channel. By using molecular modeling and simulations, we show that the protonated state of K245 (K245^+^) favors the open fenestration state and, symmetrically, that the open fenestration state favors the protonated state of the lysine residue. We show that the channel can be completely blocked by Prozac, which is known to induce fenestration opening in TREK-2. K245 protonation and fenestration aperture have an additive effect on the conductance of the channel. The opening of the fenestrations with K245^+^ increases the entrance of lipids into the selectivity filter, blocking the channel. At the same time, the protonation of K245 introduces electrostatic potential energy barriers to ion entrance. We computed the free energy profiles of ion penetration into the channel in different fenestration and K245 protonation states, to show that the effects of the two transformations are summed up, leading to maximum channel blocking. Estimated rates of ion transport are in qualitative agreement with experimental results and support the hypothesis that the most important barrier for ion transport under K245^+^ and open fenestration conditions is the entrance of the ions into the channel.

## 1. Introduction

Two-pore domain potassium (K_2_P) channel family members, also known as leak K^+^ channels, play a pivotal role in maintaining background conductance at the plasma membrane stabilizing the resting membrane potential [1]. Fifteen mammalian genes encoding K_2_P channels were identified, which are classified into six subfamilies, according to their sequence identity and functional properties [2]: TWIK, THIK, TREK, TASK, TRESK, and TALK. K_2_P channels exhibit one of the most versatile patterns of regulation known for any class of ion channels [3,4]. They respond to a wide range of regulatory factors, which include extra- and intracellular pH (pH_o_ and pH_i_) [5,6,7], temperature [8], membrane stretch [9], polyunsaturated fatty acids [10,11], and pharmacological compounds (e.g., volatile anesthetics [12] or antidepressants [13,14]). Currently, the crystallization of 20 different structures of these channels from the TREK, TWIK, and TASK subfamilies has considerably increased our comprehension of the structure and function of K_2_P channels (Table S1).

The K_2_P channels assemble as dimers with two pore-forming domains (PD1 and PD2) and four transmembrane helices (TM1–TM4) per subunit. The inner pore-lining helices TM2 and TM4 are in quasi-oblique position below the selectivity filter (SF) with respect to the membrane, while the outer helices TM1 and TM3 are located vertically. Two side-openings, called fenestrations, were resolved in K_2_P channel crystallographic structures [15]. These portals connect the lipid membrane with the central cavity below the SF. In the TWIK-1 structure (Protein Data Bank code (PDB): 3UKM; Table S1), Miller et al. [15] suggested that these fenestrations can be occupied by lipid acyl chains, which would interrupt the ion flux at the central cavity. Subsequently, a high-resolution TRAAK structure [16] (PDB: 4I9W; Table S1) showed that the fenestrations could coexist in open or closed states, depending on the TM4 down or up movements, respectively. Brohawn et al. [16,17] postulated that the TM4 helix in up-state corresponds to the conductive state in TRAAK. In contrast, the down-state allows lipids protruding from the fenestration into the central cavity blocking the passage of the ions. The TREK-2 structure (PDB: 4XDK; Table S1) co-crystallized with the inhibitor norfluoxetine (NFX) by Dong et al. [14] is consistent with Brohawn’s hypothesis, where the down conformation primarily corresponds to the closed channel. Regarding the SF in TREK-2, the ion occupancy decreased from four to three potassium when the TM4 helix was found in the down-state, suggesting that gating may occur at the SF level.

Eight of the 15 mammalian K_2_P channels respond prominently to changes in extra- or intracellular H^+^ concentration. They are related with numerous physiological functions, from hormone secretion to central respiratory adjustment [6,18]. TASK-2 or TWIK-related acid-sensitive K^+^ channel 2, belongs to the TALK subfamily sharing between 33% and 36% sequence identity with other TALK members (TALK-1 and TALK-2/TASK-4). It was identified in the human kidney as an outward rectifying K^+^ channel at physiological potassium concentrations [19]. TASK-2 plays important roles in cell volume regulation, bicarbonate reabsorption in the proximal tubule of the nephron, and CO_2_ chemosensing in neurons of the retrotrapezoid nucleus [20]. More recently, roles for TASK-2 in K^+^ recycling in cochlear outer sulcus cells [21] and in the process of anion secretion initiated by cyclic adenosine monophosphate (cAMP) in intestinal epithelium [22] were also reported.

The TASK-2 channel is activated by extracellular alkalinization [19]. The pH_o_ sensor in TASK-2 is an arginine residue (R224) located at the outermost portion of the TM4 helix and near to PD2. This pH_o_ sensor is protonated at acidic pH_o_, affecting the electrostatic potential of the SF, the ion occupancy and, therefore, the gating [23]. The basic residues in this position are conserved within the TALK subfamily, suggesting a unified alkaline pH_o_-gating mechanism for the TALK clade [23]. TASK-2 can be also opened by intracellular alkalization, mediated by a neutralization of the residue K245 [7]. This pH_i_ sensor is positioned at the end of the TM4 helix. Mutation in K245 by alanine or cysteine remove the dependence of pH_i_ in the TASK-2 channel [7]. Our group built a molecular model of TASK-2 based on the TRAAK structure [1], in order to estimate the relation between the Brohawn’s hypothesis [16,17] and the pH_i_ mechanism. Based on this model, we proposed that a neutral K245 would favor the proximity of TM2 and TM4 by hydrophobic interactions that close the TASK-2 fenestrations, leading to channel opening. On the other hand, a protonated K245 at acidic pH_i_ would favor a TM4 change from up- to down-state, opening the side fenestrations and closing the channel. Intra- and extracellular pH-gated mechanisms in TASK-2 are independent [7]. The pH_o_ modulation is extracellular K^+^- and voltage-dependent; instead, pH_i_ gating is not affected by either factor [7]. The pH_o_ dependence controls the gate of TASK-2 in a mechanism reminiscent to C-type gating at the SF level, whereas the pH_i_ mechanism remains unclear.

The main goal of this investigation was to study the molecular blockage mechanism that protonated K245 (K245^+^) exerts in TASK-2 gating and its relationship to the side fenestrations, using computational methodologies. The tridimensional topologies of K_2_P channels are quite similar, supporting the premise that the crystallographic K_2_P data can be used to build molecular models of the TASK-2 channel to accomplish our research goal.

Our results show that, firstly, K245^+^ in an up-state might disrupt the TASK-2 channel function, altering the SF. Secondly, during molecular dynamics (MD) simulations, K245^+^ precluded closure of the fenestrations and the transformation of the channel to the up-state. Thirdly, although closure of TASK-2 channel fenestrations was only observed by MD simulations, we validated the existence of the fenestrations by blocking the TASK-2 channel with NFX (half maximal inhibitory concentration (IC_50_) = 17 ± 5 µM). Fourthly, NFX can occupy a similar position in the fenestrations of the TASK-2 channel as in TREK-2 structure. Fifthly, protonation/deprotonation events of K245 take place in the down-state. Sixthly, once the fenestrations are opened and K245 is protonated, the current block of the TASK-2 channel could occur via a positive electrostatic potential effect of K245^+^. In addition, K245^+^ could induce a higher number of lipid molecules protruding trough the side fenestrations. Seventhly, the TASK-2 channel could exist in three states in terms of the intracellular pH sensing mechanism: (i) in the up-state and K245 residue neutral (K245^0^); (ii) in the down-state and K245 residue neutral; and (iii) in the down-state and K245 protonated. Lastly, the most conductive state of TASK-2 is the first, whereas the second state is about 1 kcal/mol more favorable than the third. We expect that these results might contribute to the rational design of molecules to interact with K245 to modulate TASK-2 channel function.

## 2. Results

### 2.1. Homology Modeling of TASK-2: Model Relaxation and Equilibration

Here, we aimed to study the effects on the conduction properties of the TASK-2 channel as a function of the aperture or closure of its fenestrations and as a function of the protonation state of K245. Homology models of the TASK-2 channel were obtained from known crystallographic structures in different fenestration states (see Section 4 for details). For each subset of templates, 150 independent models were built with Modeller [24].

Six crystal structures with both fenestrations open (OO) selected from TREK-2 (PDB identifiers 4XDK, 4XDJ, and 4XDL) and TASK-1 (PDB: 6RV2, 6RV3, and 6RV4) channels (Figure S1A) were used to build TASK-2 homology models with OO fenestrations. The final TASK-2 homology model using TREK-2 structures as templates was named T2Tre2OO, while the TASK-2 model using TASK-1 structures as templates was named T2Tas1OO.

Models of the TASK-2 channel with both fenestrations closed (CC) were built from templates obtained from TREK-1 structures (4TWK, 6CQ8, 6CQ6, 6CQ9) and TRAAK (4WFE, 4WFG). The subset of models built using TREK-1 structures was named T2Tre1CC. The subset of models built using TRAAK structures was named T2TraaCC.

**Figure 1:**
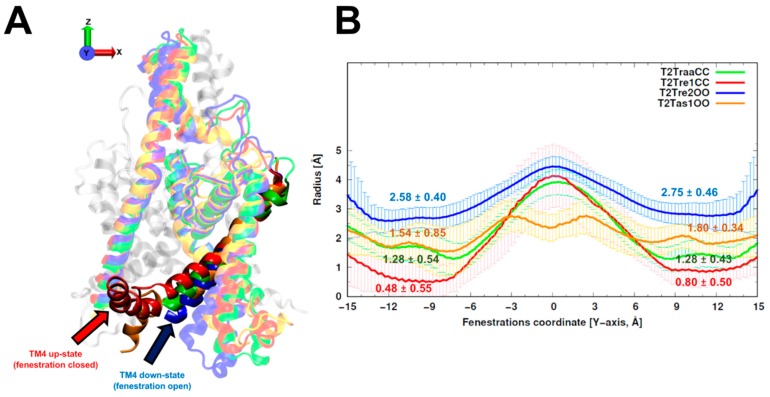
Comparison of the fenestration dimensions between TASK-2 homology models. (**A**) Structural alignment of the best T2Tre2OO model (blue), T2Tre1CC model (red), T2TaskCC model (yellow), and T2TraaCC model (green). The TM4 helix of one monomer is shown by an arrow indicating the up- or down-state; (**B**) The average radius of each fenestration (± standard deviation) from 150 homology models of the TASK-2 channel calculated with the HOLE program [26]. The fenestrations are disposed in the *X*-axis [−15, −6] Å and [6, 15] Å, while the central cavity is located at [−5, 5] Å approximately. The numbers over each plot represents the bottleneck radius in each fenestration.

The average and standard deviations of fenestration radii for the 150 models obtained with different template sets are shown in Figure 1. The TASK-2 models based on the recently solved TASK-1 structures (T2Tas1OO) were barely over the cutoff (1.6 Å) proposed by Jorgensen et al. [25] to consider the fenestrations open. As expected, the radius of the fenestrations in the model of the open state (T2Tre2OO) was significantly larger than that for the models of the closed fenestration states (T2Tre1CC and T2TraaCC). Indeed, the bottleneck radii of T2Tre2OO models were three- and five-fold (2.75 and 2.58 Å) greater than those of the T2Tre1CC models (0.80 and 0.48 Å). The T2TraaCC and T2Tas1OO models showed an intermediate state of closure/opening for both fenestrations compared with the other models. The models representing the extremes of aperture and closure of the fenestrations (T2Tre1CC and T2Tre2OO) were selected for further analysis through molecular dynamics (MD) simulations in different K245 protonation states.

Initially, 100-ns MD simulations were performed in triplicate for each system (T2Tre1CC and T2Tre2OO) and with the two possible protonation states of K245. The models were named T2Tre1CC-K245^+^ and T2Tre1CC-K245^0^ for protonated and neutral K245 states of the channel with the closed fenestrations, and T2Tre2OO-K245^+^ and T2Tre2OO-K245^0^ for the pronated and neutral K245 states of the channel with open fenestrations.

The overall variability of the models along the 100-ns MD simulations was evaluated by computing the root-mean-square deviations (RMSDs) relative to the initial structures. The two models with open fenestrations (T2Tre2OO-K245^+^ and T2Tre2OO-K245^0^) and the model of the neutral closed fenestration state (T2Tre1CC-K245^0^) displayed RMSDs systematically smaller than about 3 Å, indicating that the structures were highly preserved (Figure S2A). The model of the closed fenestration state and K245 protonated (T2Tre1CC-K245^+^), on the other hand, displayed larger fluctuations and, although the RMSDs smaller than 5 Å did not suggest major structural changes, some fluctuations not observed for the other models were present.

**Figure 2:**
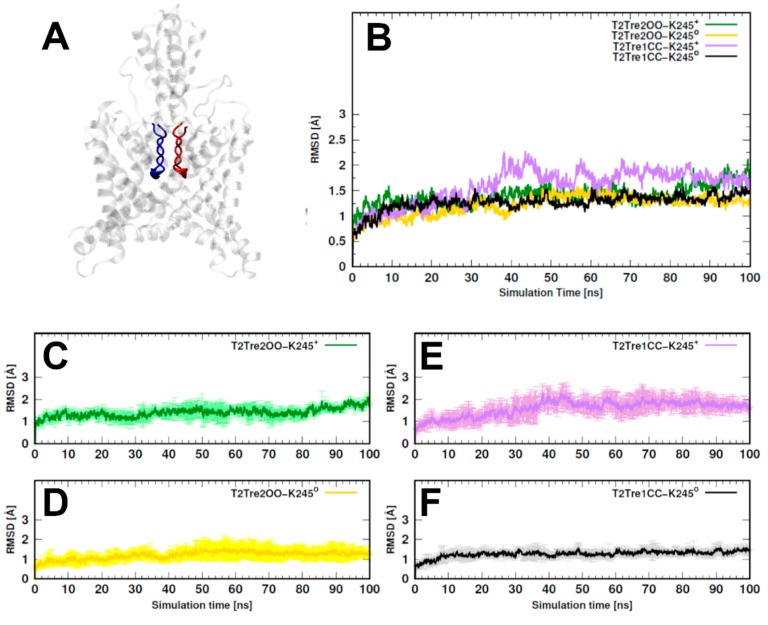
Root Mean Square Deviation (RMSD) values evaluated in the selectivity filter of TASK-2 homology models during molecular dynamics (MD) simulations. The time dependence of the RMSD values calculated over the residues 97–103 and 203–208 for both monomers. (**A**) An example of T2Tre1CC showing the selectivity filter of both monomers in red and blue colors; the transparent gray region was not considered in this RMSD calculation; (**B**) RMSD averaged over three replicas for all systems. Averages and standard deviations of RMSD values for T2Tre2OO-K245^+^ in green color are depicted in (**C**), and those for T2Tre2OO-K245^0^ in yellow are shown in (**D**); Correspondingly to (**C**,**D**), values for the T2Tre1CC model are shown in (**E**,**F**).

In Figure 2, we show the RMSDs of the residues of the selectivity filter (residues 97–103 and 203–208, as shown in Figure 2A), which displayed a similar trend as observed for the entire structures, but with smaller deviations. Effectively, for the two open states and for the neutral closed state, the channel residues preserved the initial structure within 2 Å RMSD (Figure 2B–D,F), while, for T2Tre1CC-K245^+^-, larger structural deviations were observed, and the RMSD variability between MD replicas was also greater for this model (Figure 2E). To avoid biases associated with the initial models used, analyses were performed in all cases for the last 50 ns of simulations.

The greater structural variability of the T2Tre1CC-K245^+^ model could be associated with disfavored hydrophobic interactions that close the fenestrations. The protonation of K245 led to increased fluctuations of the TM4 helix in both T2Tre2OO-K245^+^ and T2Tre1CC-K245^+^ (Figure S3A) and, in the case of the closed fenestration model, increased fluctuations were observed also in the TM2 helix. The TM2 helix interacts with TM4 (K245 included) only if the fenestrations are closed. Therefore, the simulations show that the protonation of K245 increases the structural fluctuations of TM4, and that these fluctuations propagate to TM2 if the fenestrations are closed. Nevertheless, we could not identify hydrogen bonds or salt bridges systematically involving the K245 residue in any system, such that these protonation effects on the fluctuations of TM2 might impede the hydrophobic interactions that close the fenestrations.

**Figure 3:**
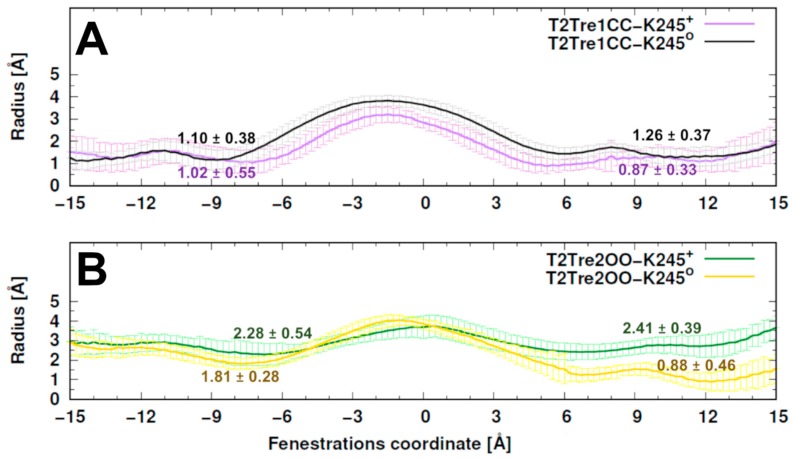
Comparing the dimensions of the fenestrations during TASK-2 MD simulations. (**A**) represents the average ± standard deviation of both fenestrations size for TASK-2 simulations (T2Tre1CC-K245^+^ in purple and T2Tre1CC-K245^0^ in black). Similar to (**A**), TASK-2 simulations at the down-state of the fenestrations are shown in (**B**) for T2Tre2OO-K245^+^ in green and T2Tre2OO-K245^0^ in yellow. The calculations were performed with the HOLE program for three replicas per system. The fenestrations are disposed in the *X*-axis [−15, −6] Å and [6, 15] Å, while the central cavity is located at [−5, 5] Å approximately. The numbers over each plot represent the bottleneck radius in each fenestration.

The radii of the fenestrations within the simulations are shown in Figure 3. The bottlenecks of closed states varied around 1 Å radius and were similar in both protonation states. A slightly smaller central cavity was found in T2TreCC-K245^+^ relative to T2Tre1CC-K245^0^ (Figure 3A). The bottlenecks for open states varied between averages of 0.88 Å and 2.41 Å. A constriction of the fenestration was observed for the neutral K245 model, mainly at the right-side fenestration, regarding the initial configuration (~2 Å in T2Tre2OO-K245^0^—Figure 3B versus Figure 1). Thus, the neutral K245 led to a partial closure of the fenestrations in the simulations starting with the open states.

The rotamers of the side chain of K245 could be classified into two distinguishable clusters, as shown in Figure S4A: (1) the ε-amino group in a pseudo-orthogonal position regarding the conduction pore, orienting the side chain toward the lipidic membrane (in cyan color), and (2) the ε-amino group in a quasi-parallel position with respect to the pore (in yellow color). Both conformers were identified in all but the T2Tre1CC-K245^+^ system, for which the K245 side chain was only found in the pseudo-orthogonal conformation (Figure S4B).

The pKa values for the TASK-2 K245 residues were estimated from the molecular environment observed in the MD simulations and are shown in Table 1. The protonation led to fluctuations in the molecular environment of K245 that, as expected, implied a greater pKa (the variations of the molecular environment in the presence of the proton should stabilize its presence, thus increasing the basicity required to remove it). This stabilization of the protonated state was stronger in the open fenestration states (pKa ≈ 8.65 and 8.98) than in the closed fenestrations (pKa ≈ 7.95 and 7.75). Therefore, the protonation of K245 is more stable with the fenestrations open. The average pKa for K245 in open-state simulations (T2Tre2OO-K245^+^ and T2Tre2OO-K245^0^) was 7.93 ± 0.98, and that in closed-state simulations (T2Tre1CC-K245^+^ and T2Tre1CC-K245^0^) was 7.34 ± 0.63. Both pKas were, within the fluctuations, consistent with the experimental pK_1/2_ of 8.0, but the data suggests that the experimental pKa is closely associated with the protonation events in the open fenestrations.

In summary, structural models were built for TASK-2 with open and closed fenestrations, and MD simulations were performed for the final models in the two possible K245 protonation states. The models displayed fenestration radii consistent with the expected aperture of the channels, but the closure of one of the fenestrations was observed in a K245 neutral state starting from an open state. The overall structures were mostly stable within the timescales of the simulations, except for an apparent instability of the TM2 helix in the closed protonated state. The simulations suggest, then, that the neutral open state and the protonated closed state of the fenestrations are somewhat unstable, and that there is a coupling between the aperture of the fenestrations and the protonation of the K245 residue.

### 2.2. Fenestration Aperture and Channel Conductivity

**Figure 4:**
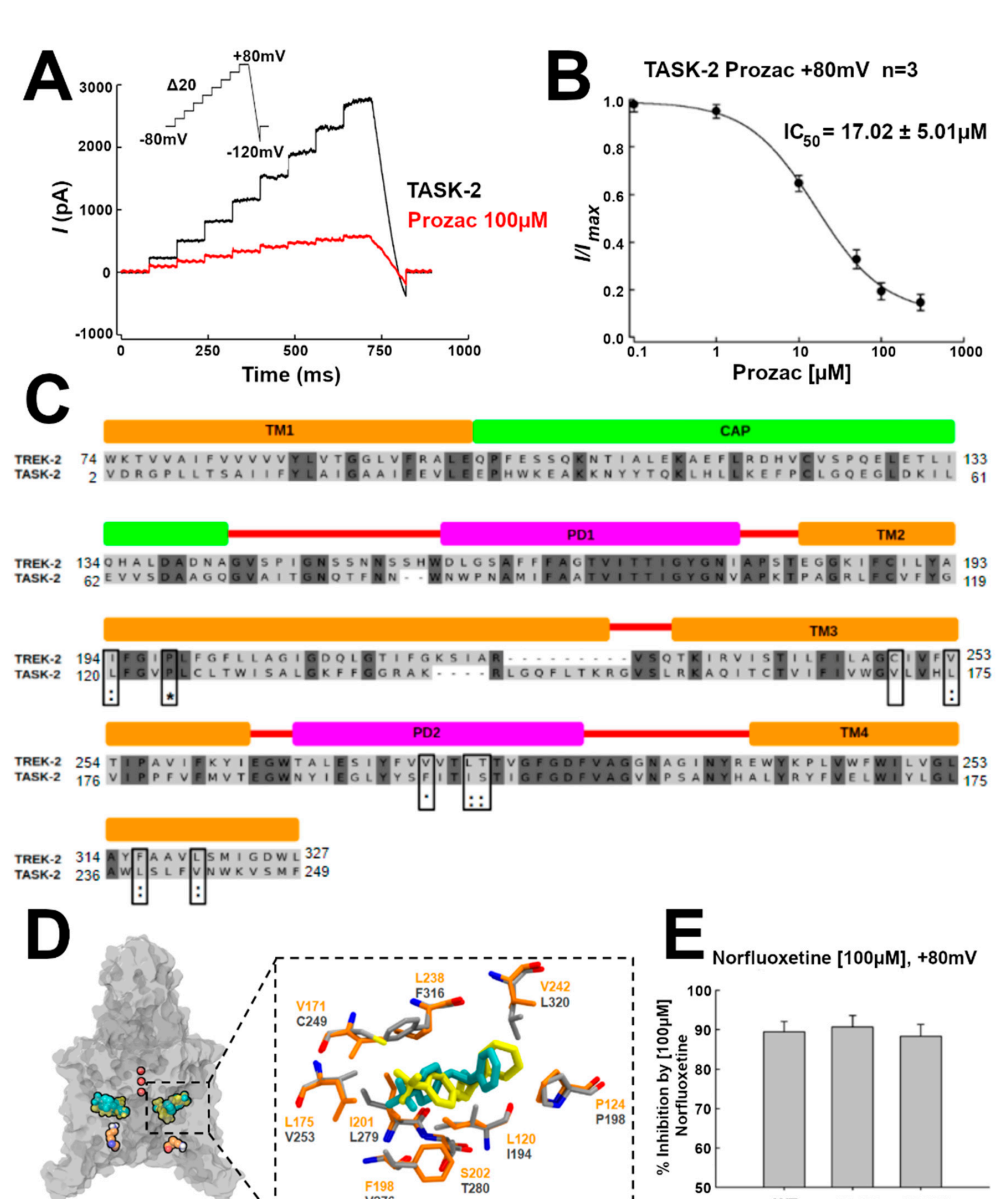
TASK-2 is blocked by Prozac. (**A**) Whole-cell recordings showing the effect of Prozac (100 µM) on TASK-2 channels. The currents were evoked using the voltage protocol from −80 to +80 mV (see inset); (**B**) Concentration–response curve with the IC_50_ value of TASK-2 inhibition by Prozac. The block was analyzed at the end of the test pulse at +80 mV; (**C**) Docking of Prozac in the T2Tre2OO structure (yellow) shows a similar position as Prozac in the TREK-2 crystal structure (cyan). Prozac binding site residues in TREK-2 are shown in gray, whereas the homologous residues in TASK-2 are in orange. The protein is shown in transparent surface representation. The ions in the selectivity filter are red. K245 residues are shown as reference in orange; (**D**) Pairwise sequence alignment between TREK-2 and TASK-2 channel [27]. Prozac binding site in TREK-2 is composed by residues of TM2, TM3, PD2, and TM4. They are conserved in TASK-2 channel. * Identical residues; **:** very conserved residues; ∙ conserved residues; (**E**) Percentage of inhibition by 100 µM Prozac of wild-type TASK-2 and two mutants of K245 residue. Results are shown as means ± Standard Error of the Mean (SEM).

The TREK-2 channel conductance is inhibited by NFX, the active metabolite of fluoxetine (Prozac). A crystallographic structure of the NFX–TREK-2 complex was described (PDB 4XDK), with NFX located in the open fenestrations [14] (Table S1). We hypothesized that, if TASK-2 can also display open fenestrations, NFX should also block the TASK-2 channel. Indeed, as shown by current traces and the IC_50_ in Figure 4A,B, respectively, NFX blocked the conductivity of TASK-2 (at pH = 7.4, where we expect that K245 is mostly protonated) [7]. Additionally, we docked NFX in the TASK-2 model with open fenestrations (T2TreOO), and found that NFX can occupy a similar position to that reported in the TREK-2 structure (Figure 4C). The norfluoxetine binding site [14] is conserved in TASK-2 except for a cysteine (C249) in TREK-2 which is replaced by V171 in TASK-2 (Figure 4C,D). Thus, a stabilization of an open fenestration state induced by NFX would inhibit the channel, consistent with the concept that a conducting structure is that with fenestrations closed.

**Figure 5:**
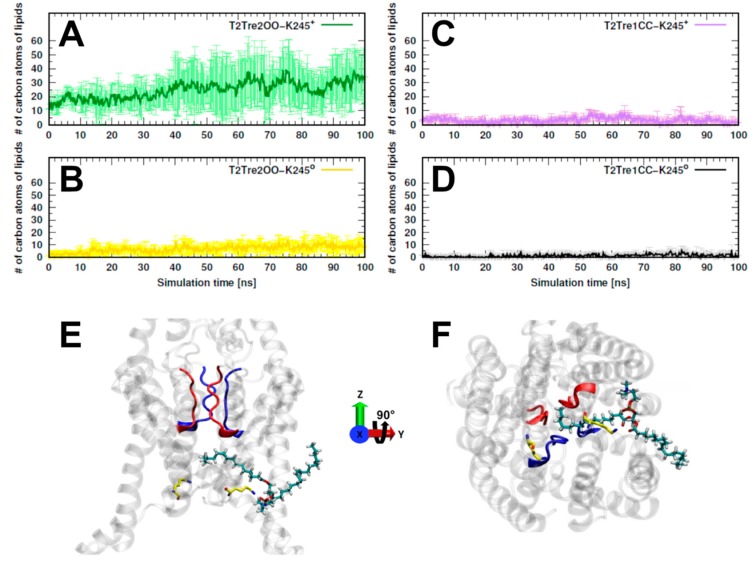
Evaluation of the lipids protruding toward the fenestrations and the central cavity during the entire simulation time. The entrance of lipids in the conduction pore and fenestrations were measured as a function of the number of carbon atoms belonging to the lipids and protruding into these regions. (**A**,**B**) The average of carbon atom number in three replicas for T2Tre2OO-K245^+^ and T2Tre2OO-K245^0^, respectively. Correspondingly to (**A**,**B**), they are shown for T2Tre1CC-K245^+^ and T2Tre1CC-K245^0^ in (**C**,**D**). The figures (**E**,**F**) represent the frontal and intracellular views of a 1-palmitoyl-2-oleoyl-sn-glycero-3-phosphocholine (POPC) lipid molecule protruding through the side fenestration in the T2Tre2OO-K245^+^ simulation. K245 is shown in yellow.

The inhibition of K_2_P channels by the opening of the fenestrations was suggested to be associated with the blocking of the passage of ions as a consequence of the penetration of the lipids into the channel [17]. We computed, then, the number of carbon atoms belonging to lipids that were found inside the fenestrations along the entire MD simulations. In Figure 5A, we see that, for T2Tre2OO-K245^+^, about 30 to 40 lipid atoms penetrated the fenestrations. For the simulation initiated with the open channel but with neutral K245 (T2Tre2OO-K245^0^), there was some insertion of the lipids (at most about 10 C atoms, Figure 5B), which was already inhibited by the partial constriction of the fenestrations (Figure 3B). For the closed fenestration simulations (T2Tre1CC-K245^+^ and T2Tre1CC-K245^0^, Figure 5C,D), the lipids could not penetrate the channel. Figure 5E,F show the lipids in the conduction pore, crossing with one hydrophobic tail through the fenestrations, and reaching the central cavity below the SF in T2Tre2OO-K245^+^ simulations. Thus, these results are consistent with a mechanism of channel blocking by lipid insertion into the open fenestrations.

### 2.3. K245 Protonation State and Channel Conductivity

The protonation of K245 appears to be coupled with the aperture of the fenestrations. In Reference [7], Sepúlveda and co-workers showed that the channel conductance is mostly unaffected by mutating this lysine residue to alanine. This indicates that the channel can exist in the closed state, which is conductive, in the neutral K245 state. Here, we studied the inhibition of the conductance by NFX in pH_i_-insensitive mutants to address the hypothesis that the fenestration can also be found open in the neutral state. Effectively, as shown in Figure 4E, the mutants TASK2-K245A and TASK2-K245V are inhibited by NFX similarly to the native TASK2, implying that NFX can bind the neutral state. Since NFX binds to the down-state, that is, when the fenestrations are open, the channel can exist with open fenestrations in the neutral K245 state. Summing up, these results indicate that, in the neutral K245 state, the channel can be found in the open and in the closed fenestration states. Our simulations provide an indication that the closed fenestration state might be more stable, as the closure of one of the fenestrations was observed in the T2Tre2OO-K245^0^ simulation (Figure 3B).

Clear evidence exists of the stability of the open fenestration state when K245 is protonated. The simulations of the T2Tre2OO-K245^+^ were stable, and the pKa of the K245 residues was the greatest, supporting the stability of the protonated state when the fenestrations are open. The average pKa computed from the two down-state (neutral and protonated) simulations was consistent with the experimental pKa, suggesting that the equilibrium of protonation and deprotonation of the lysine occurs in the open state (Table 1).

The demonstration of the existence of a significant population, or not, of the closed fenestrations with the protonated K245 is, however, difficult. Our equilibrium simulations indicated that there are significant fluctuations of the structure in these conditions (Figure 2B), suggesting a possible instability, although the opening of the fenestrations was not observed in the timescale of the simulations (Figure 3). Experimentally, it is clear that the channel is inhibited at pH_i_ ≲ 8.0 [7], when K245 is expected to be protonated. This inhibition can be direct via the introduction of electrostatic repulsion, but also mediated by the shift in equilibrium from the closed to the open state upon protonation.

**Figure 6:**
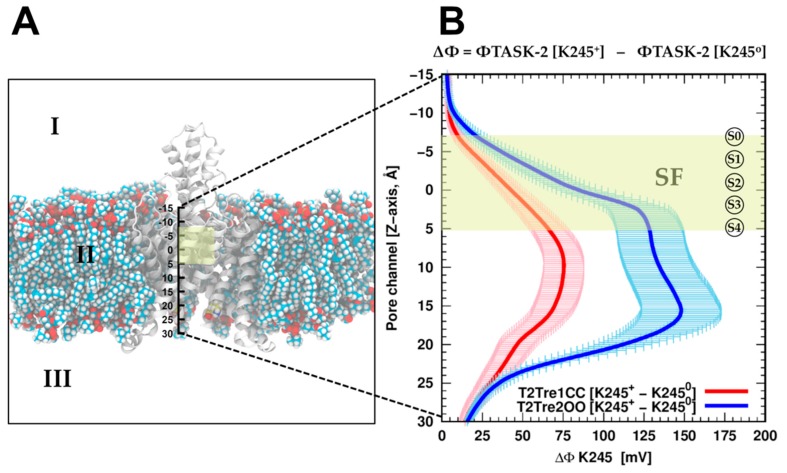
Electrostatic potential difference (ΔΦ) of K245 in TASK-2 systems. The ΔΦ was computed for protonated TASK-2 systems by solving the Poisson–Boltzmann equation with APBS software through the last 50 ns of the trajectory. Both the TASK-2 channel and the lipid membrane exported from the MD simulations were used explicitly in the calculation. The dielectric constant was set at ε = 2 for zone II (protein and membrane) and ε = 80 for zone I and III, corresponding to an implicit solvent with an ionic concentration of 150 mM of KCl. The ΔΦ was measured along the *Z*-axis between the coordinates [−15, 30] Å represented in (**A**). This range in *Z*-axis corresponds to the extracellular region of the channel Z < −15 Å, and the end of the inner helices TM2 and TM4 in the intracellular space is represented by Z = 30 Å; (**B**) ΔΦ plot for TASK-2 based on TREK-1 and TREK-2 subsets representing the closed and open states of the fenestrations in red and blue colors, respectively. ΔΦ is the subtraction of the electrostatic potential (Φ) of the system with K245 protonated from the Φ of the system with K245 neutral at the *Z*-axis [−15, 30] Å. The selectivity filter region (shown in a yellow rectangle) comprises the coordinates from S0 at Z ≈ −7 Å until S4 at Z ≈ 5 Å. The site in the central cavity (Scav) is located at Z ≈ 10 Å approximately.

To evaluate if the protonation of K245 can introduce a direct inhibition of the conductance by direct electrostatic interactions, the effect of K245 protonation on the electrostatic potential of the channel was calculated. Adding the proton K245 increased the electrostatic potential, as expected and shown in Figure 6B, for the channel in both the open fenestration state and the closed fenestration state. Therefore, adding the proton works against the presence of potassium ions in the channel in both states from the point of view of direct electrostatic interactions. However, this effect is much greater when the fenestrations are open than when they are closed. This difference stems from the orientation of the K245 side chains in each fenestration state. As shown in Figure S4A, in the closed state, the side chains of the K245 residues cannot adopt an orientation in which it points inward into the channel. Thus, from the analysis of direct electrostatic interactions, the inhibitory effect of protonating the K245 side chains is expected to be greater with the fenestrations open. From this point of view, the inhibition of the conductance at low pH_i_ results from the coupled effects of protonation and fenestration opening.

Therefore, assuming that this electrostatic repulsion impairs the entrance of the ions into the channel, the K245 protonation would decrease the conductivity of the channels in the open state of the fenestrations more than in the closed state. In other words, the sensitivity of the channel in its closed fenestration state to the protonation of K245 is relatively small. This must be combined with the fact that the pKa of K245 in the closed fenestration state is lower and, thus, it is less likely that this residue is protonated in this state. Summing up, we expect that protonation is less common for the closed fenestration state and, when it occurs, it affects the electrostatic potential of the channel less than for the open state, favoring ion conduction. By contrast, K245 is found more frequently in the protonated state if the fenestrations are open and, additionally, the effect of protonation is relatively large on the electrostatic potential of the channel, with both effects leading to decreased ion transport.

### 2.4. Reaction Rates from Free Energy Profiles of Ion Transport

The above analysis assumes that the major barrier for conduction, under K245^+^ and open fenestration conditions, is the occupancy of the permeation pathway by the ions. This assumption can be supported by molecular dynamics simulations. We performed free energy calculations of K^+^ entrance into the channels in open and closed fenestrations and in K245 protonated and deprotonated states. Potentials of mean force (PMFs) were obtained for the passage of the ions from the cytosolic solution to the central cavity of the channel, as a function of the fenestration state and K245 charge.

**Figure 7:**
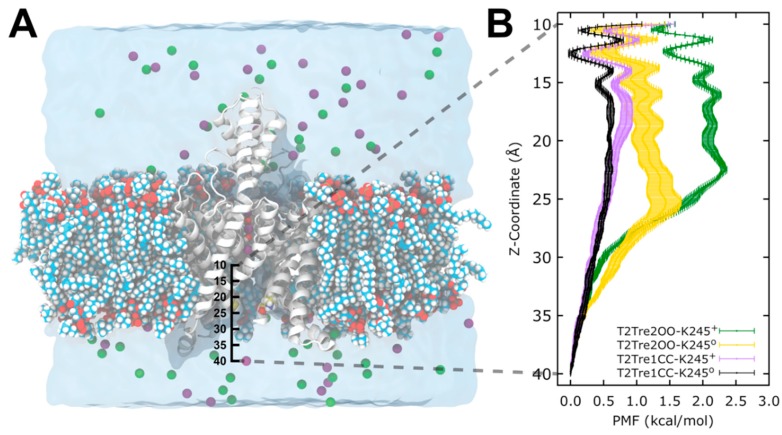
Potential of mean force (PMF) of K245 in TASK-2 channel. The PMF calculations were measured as is depicted in (**A**), translocating one K^+^ ion from the cytosolic region (coordinate *Z* = 40 Å) to the central cavity (coordinate *Z* = 10 Å) below to the selectivity filter in the *Z*-axis for the four configurations; (**B**) The free energy barriers calculated along the conduction pore in the *Z*-axis [10, 40] Å.

In Figure 7, we see that, for the channel with closed fenestrations (black and violet curves), the major barriers for ion conductance were within the central cavity of the channel (*Z* < 20 Å), while, in the open state, there was an additional and dominant barrier at the channel entrance from the intracellular side (*Z* ~ 25 Å). This dominant barrier was further increased with the protonation of the K245 residue (green plot).

From the PMFs of Figure 7, it is possible to obtain estimates of the relative ion permeation rates, assuming that the maximum barriers sampled are determinant. In a first approximation, it is possible to use transition state theory to compute the rates of ion transport from the highest free energy barrier. The relative rates of reaction of two mechanisms can be computed by

(1) $$\frac{k_{1}}{k_{2}}=e^{\left(\Delta G_{1}-\Delta G_{2}\right)/RT},$$

where *k*_2_ and *k*_1_ are the two rate constants, and Δ*G*_2_ and Δ*G*_1_ are the free energy barriers computed relative to the same reference state.

**Figure 8:**
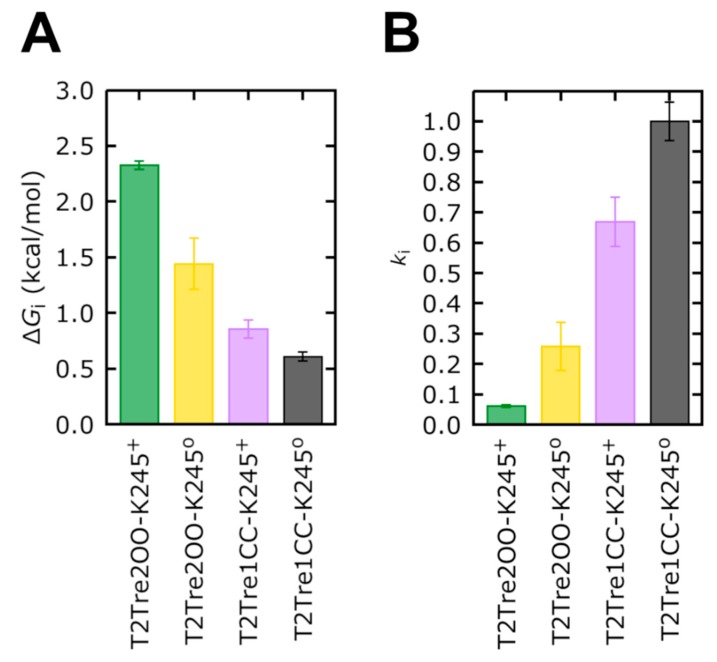
Free energy and rate constants of K245 in TASK-2 channel. (**A**) The free energy ΔG_i_ cost and (**B**) the kinetic rate constant *k*i were obtained from the highest barrier in the PMF simulations. *k*i was calculated from the Equation (1).

Firstly, the free energy profiles of the closed fenestration states (Figure 7B) differed by at most 0.25 kcal/mol (Figure 8A). This free energy barrier difference implies rates of ion transport which differed between the two closed states by at most a factor of ~1.5 (Figure 8B).

The opening of the fenestrations introduces greater barriers, however. Here, considering as the reference state the ion in the cytosolic solution (reaction coordinate *Z* = 40 Å), we obtained that the highest barrier observed for the channel with open fenestrations and a neutral K245 (yellow curve in Figure 7B) was of 1.44 kcal/mol, which is 0.84 kcal/mol greater than the maximum free energy barrier of the system with the fenestrations in the closed state with neutral K245 (black bar in Figure 8A). This implies that ion transport through T2Tre2OO-K245^0^ is ~4.0 times slower than for T2Tre1CC-K245^0^ due to this barrier (Figure 8B). Thus, the opening of the fenestrations reduces the ion transport rate by about four times, according to the PMFs obtained here. The opening of the fenestrations, when coupled to the protonation of the K245 residue, led to the highest PMF values shown in Figure 7B. This PMF calculation displays a maximum free energy barrier of 2.32 kcal/mol (Figure 8A), which is 1.72 kcal/mol greater than the barrier of T2Tre1CC-K245^0^. The transport rate of the system with fenestrations closed in the deprotonated state is, thus, ~16 times greater than the system with the open fenestrations in the protonated state (Figure 8B).

The combined effects of protonation and fenestration opening result, therefore, in a reduction of channel conductance (as measured by the ratio of transport rates) of about 16-fold. The experimental inhibition of the conductance obtained by reducing the pH from −9.5 to −6.5 is of about 10 times for the wild-type TASK2 [7], which is similar to our result. Therefore, the inhibition observed experimentally by varying the pH appears to be the combined effect of protonation and fenestration opening.

In summary, the free energy profiles of ion permeation from the cytosolic solution to the channel central cavity predict rates of ion transport in qualitative agreement with experimental observations. These results support the hypothesis that the major barrier for conductance is the entrance of ions in the channel, which is modulated by the fenestration aperture state and charge of K245. These effects cannot be easily decoupled in the experimental set-up as the protonation of K245 induces the opening of the fenestrations.

## 3. Discussion

TASK-2 is a member of the K_2_P channel family activated by alkaline intracellular [7] and extracellular pH [5,23]. The pH_o_-insensitive TASK-2-R224A mutant is not altered in its pH_i_ dependence, suggesting that the pH_i_ dependence of TASK-2 occurs independently of the SF gating responsible for pH_o_ dependence [7]. Neutralization of a lysine residue (K245) located at the C-terminal end of TM4 abolishes gating by pH_i_ [7]. Previously, our group hypothesized that a neutral K245 favors the hydrophobic interactions that close the fenestrations, but K245^+^ would preclude the fenestration closing [1].

Here, 20 K_2_P channels structures were crystallized from the TREK, TWIK, and TASK subfamilies. They served as blueprints in this work to model the TASK-2 channel with closed and open fenestrations (Figure 1). We propose that K245^+^ in a closed state of the fenestrations might disrupt the TASK-2 channel function, altering the SF. A clear insight of stability loss was observed in the RMSD analysis of the selectivity filter of T2Tre1CC-K245^+^ molecular dynamics simulation (Figure 2). Previously, it was observed that activators of K_2_P channels stimulate function by reducing the dynamics of the SF [28]. Conversely, a decrease in pH_i_ will protonate the K245 residue, promoting the dynamics of the SF—in a closed state of the fenestrations—and moving TASK-2 to a non-conducting channel.

A stability loss was observed also in the RMSD analysis of the whole protein (Figure S2) in T2Tre1CC-K245^+^ MD simulations. Root-mean-square fluctuation (RMSF) analysis (Figure S3) and this could explain how the protonation of K245 at TM4 disrupts the selectivity filter. A charged K245 alters the apposition of TM4 and TM2, inducing the movement on both segments. These fluctuations were observed in T2Tre1CC-K245^+^ MD simulations and could alter the SF which is directly above TM2 and TM4. An RMSF increase can also be observed in the TM4 segment in the MD simulations based on the T2Tre2OO model. However, the T2Tre2OO model does not include the C-terminal region, and the reason for these fluctuations could be the shortened model, ending exactly with TM4.

The hypothesis that K245^+^ would preclude the fenestration closing [1] is supported by comparison of the dimensions of the fenestrations during TASK-2 MD simulations (Figure 3). Similar to the 100-ns MD simulations published earlier for TREK-2 [14], TASK-1 [29,30], or TWIK-1 [25] channels, the T2Tre2OO model changed into the up-state during MD simulations and the side fenestrations closed but only in the T2Tre2OO-K245^o^ simulations (Figure 3B, Video S1). The fenestration closing was prevented during T2Tre2OO-K245^+^ MD simulations. The bottleneck radius of the fenestrations significantly decreased in the T2Tre2OO-K245^0^ MD simulations, while K245^+^ stabilized the bottleneck radius of the side fenestrations at 2.35 Å over the 100-ns MD simulations (Figure 3B). Thus, K245^+^ precludes closure of the side fenestrations and the transformation of the channel to the up-state. Nevertheless, closure of TASK-2 channels fenestrations was only observed here by MD simulations. Our model must be tested in the future to demonstrate if TASK-2 channels gate in a similar way to other K_2_P channel subfamilies and whether they physiologically change into an up-state-like conformation.

To validate the presence of open fenestrations in TASK-2 channels, we used electrophysiological measurements applying norfluoxetine to the channels. NFX is the active metabolite of fluoxetine (Prozac) and binds within intramembrane fenestrations of TREK-2 channels [14]. McClenaghan et al. [31] reported that the activation by membrane stretch but not by pH_i_ produces a loss of NFX sensitivity in the TREK-2 channel. NFX also blocks TASK-2 with an IC_50_ = 17 ± 5 µM (Figure 4B). A docking simulation of NFX in T2Tre2OO model shows that NFX can occupy a similar position in the fenestrations of the TASK-2 channel as it does in the TREK-2 structure (Figure 4C). However, mutants should be done in the future in the residues highlighted with black boxes in Figure 4D to determine if NFX binds in TASK-2 in a homologous pocket of the TREK-2 channel. We measured also the inhibition by NFX in two functional mutants of the TASK-2 channel (TASK2-K245A and TASK2-K245V) which are insensitive to pH_i_ [7], because the 245th residue is not titratable in the mutants. Both mutants exhibit the same inhibition by NFX as the wild-type TASK-2 channel (Figure 4E), suggesting that a deprotonated state of the channel could exist also with open fenestrations. In fact, we suggest that the protonation/deprotonation events of K245 take place with open fenestrations. The average of the predicted pK_a_ values for T2Tre2OO simulations (T2Tre2OO-K245^+^ and T2Tre2OO-K245^0^) was 7.93 ± 0.98 units which is similar to the reported pK_1/2_ of K245 in the TASK-2 channel [7] (~8 units) (Table 1).

Once the fenestrations are opened and K245 is protonated, the current block of TASK-2 channels could occur in two different ways which are not mutually exclusive: (1) K245^+^ generates a positive electrostatic potential (ΔΦ) distribution within the ion pathway (Figure 6) preventing the ion flux, or (2) K245^+^ induces a higher number of lipid molecules protruding trough the side fenestrations, toward the central cavity of the channel, blocking physically the flux (Figure 5). Positive potential imparted upon the conducting pore blocking the K^+^ ion occupation is a common mechanism of K_2_P channel pH sensors [5,32] whereas, in the TRAAK channel structure, in the open state of the fenestrations, lipids accessing the internal cavity through these openings prevent ion occupancy [17].

**Figure 9:**
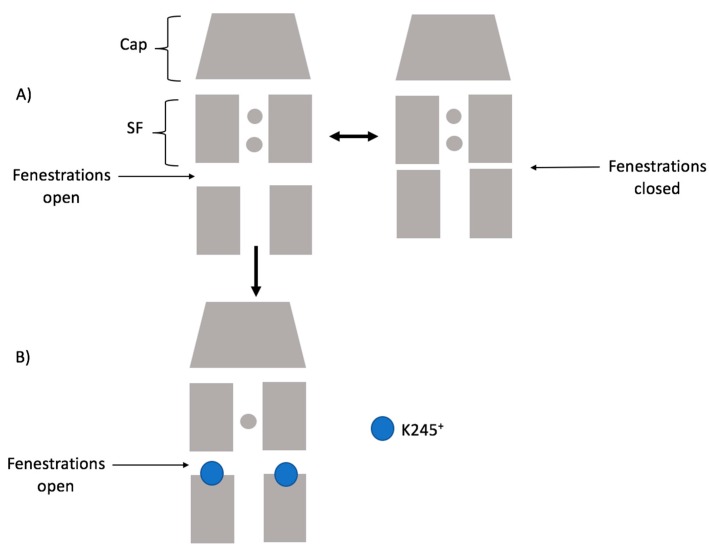
K245^+^ would preclude the fenestration closing in TASK-2 from the down-state. (**A**) The channel could switch from a down-state to an up-state; (**B**) In the down-state, K245 protonates and prevents K^+^ flux.

Considering our results, the scheme below (Figure 9) summarizes how K245^+^ would preclude the fenestration closing in TASK-2. Firstly, the channel could switch from a down-state to an up-state. Secondly, in the down-state, K245 protonates and prevents K^+^ flux. Under this scheme, the TASK-2 channel could exist in three states in terms of the intracellular pH sensing mechanism: TASK-2 with closed fenestrations and a neutral K245 residue (T2…CC-K245^0^), TASK-2 with open fenestrations and a neutral K245 residue (T2…OO-K245^0^), and TASK-2 with open fenestrations and a charged K245 residue (T2…OO-K245^+^). Figure 7 and Figure 8 show that ion flux in the T2…OO-K245^+^ system is about 1 kcal/mol less favorable than in T2…OO-K245^0^. The most conductive state of TASK-2 is T2…CC-K245^0^. This is in agreement with Brohawn et al.’s [16,17] hypothesis. They proposed that the closed state of the fenestrations is the conductive state of the K_2_P channels. Also, Brennecke and de Groot [33] studied—using molecular dynamics simulations—the conductance of the K_2_P channel TREK-2 and found that the down-state is less conductive than the up-state. We did not include the T2…CC-K245^+^ in our scheme because the closed channel is unstable if protonated (Figure 2A).

## 4. Materials and Methods

### 4.1. Homology Modeling

To date, 20 crystallographic structures of K_2_P channels are known (shown in Table S1). However, the tridimensional structure of the TASK-2 channel remains to be solved. With the purpose of studying the pH_i_ sensor of TASK-2 at a molecular level, it is crucial to have a tridimensional structure as a starting point. Homology modeling provides a way to build molecular models using templates from available data in the Protein Data Bank [34]. Two critical steps in the homology modeling protocol [35] are (1) to identify the appropriate templates, and (2) to perform the best possible alignment between the target (TASK-2 channel) and template sequences.

There are no members of the TALK subfamily solved by crystallography or cryogenic electron microscopy, and TASK-2 shares only 24%–29% sequence identity with the other 20 putative templates (belonging to TREK, TWIK, and TASK subfamilies of K_2_P channels). This low sequence identity with several possible templates is a non-trivial modeling problem. Some studies revealed that the use of multiple templates improved the quality of the models compared with the classical one-template protocol [36], by including more structural information from the reported divergences between reference structures (templates). Twenty crystallographic structures of K_2_P channels were downloaded from PDB and categorized according to the information depicted in Table S1. Two important factors to select the templates are the evolutive (sequence identity) and the structural similarity between them. The evolutive relation consists of selecting only structures belonging to the same type of channel, and the structural criteria are measured according to some attributes, like the fenestration state (open or closed), the cap orientation (domain or non-domain swapped), the completeness of the structure, and the root-mean-square deviation (RMSD) between the templates. According to the cutoff of 1.6 Å of the bottleneck radii reported by Jorgensen et al. [25], the fenestrations of each crystallographic structure were classified into open (>1.6 Å) or closed (≤1.6 Å) states, calculated with the HOLE program [26]. Four subsets of K_2_P channels templates were selected to build the TASK-2 homology models: TREK-2 (PDB: 4XDJ, 4XDK, and 4XDL) and TASK-1 (6RV2 to 6RV4) with both fenestrations opened (OO), and TREK-1 (PDB: 6CQ9, 6CQ8, 6CQ6, 4TWK) and TRAAK (PDB: 4WFE, 4WFG) with both fenestrations closed (CC). The TASK-2 homology models built starting from these template subsets were named T2Tre2OO, T2Tas1OO, T2Tre1CC, and T2TraaCC, respectively. Using each subset, 150 homology models of TASK-2 channel were generated with the Modeller program v9.18 [24], using the multiple sequence alignment proposed by Brohawn et al. [27]. TASK-2 models were ranked according to the Discrete Optimized Protein Energy (DOPE) potential score of Modeller. Then, the best five models per subset (T2Tre2OO, T2Tre1CC, T2TraaCC, and T2Tas1CC) were re-ranked by assessing the stereochemistry of the structures with the Procheck tool [37].

The HOLE algorithm [26] allows evaluating the dimensions of the cavities inside the channel. The radius of the fenestrations was measured over the 150 homology models generated for T2Tre2OO, T2Tas1OO, T2Tre1CC, and T2TraaCC. All TASK-2 models were aligned positioning the pore of the channel in the *Z*-axis, orthogonal to the membrane situated in the *X–Y* plane and the fenestrations along the *Y*-axis. The size of the fenestrations was measured every 0.25 Å in the *Y*-axis with the HOLE program [26] in each structure and averaged for each subset. The cutoff of 1.6 Å of the bottleneck radii reported by Jorgensen et al. [25] was used to discriminate between closed or open fenestrations in TASK-2 models.

### 4.2. Molecular Dynamics

The molecular dynamics simulations afford a double alternative aimed at improving the quality of the atomistic models nearest to the realistic system. In addition, they allow us to evaluate the dynamic process in terms of the molecular interactions between K245 and its microenvironment.

Due to the computational cost implied in performing all-atom simulations of membrane proteins (>70,000 atoms) only the models based on two subsets of templates were selected for MD simulations: T2Tre2OO and T2Tre1CC. The best model ranked by the DOPE score and Procheck of each subset was used as a starting point to perform MD simulations in the NAMD program [38,39] using the CHARMM force-field [40]. The Dowser program [41] was employed in order to fill the internal cavities of the channel with water molecules based on an energetics effect. Then, Helmut Grubmϋller’s SOLVATE program was used to fill the empty space within the pore and side fenestrations. Subsequently, the two TASK-2 systems (T2Tre2OO and T2Tre1CC) were embedded into a POPC bilayer membrane and solvated in a water box of TIP3P molecules with periodic boundary conditions and ionized with 150 mM KCl. Once the all-atom systems were built, they were duplicated with the residue K245 protonated (K245^+^) and neutral (K245^0^) in both chains, which were calledT2Tre2OO-K245^+^, T2Tre2OO-K245^0^, T2Tre1CC-K245^+^, and T2Tre1CC-K245^0^. The four systems were subjected to 20 ns of isothermal–isobaric (NPT, 1 atm and 300 K) simulation, where all atoms except the lipid tails were fixed, inducing the appropriate disorder in the bilayer membrane. Afterward, an energy minimization of 10,000 steps was carried out, followed by an equilibration of three consecutive MD simulations of 5 ns each, reducing the secondary structure restraints applied to the alpha-helix of the protein from 1.0 to 0.5 and 0.25 kcal·mol^−1^·Å^−2^. Finally, three replicas of MD simulations were generated for each system corresponding to 100 ns of an NPT production simulation without restraints, allowing the protein to adapt to the microenvironment. 

The RMSD of the heavy atoms was used to analyze the position of both the TASK-2 residues (2–249) and the residues that form the selectivity filter (97–103 and 203–208) in the production simulations as a function of time. Here, the RMSD values of each replica were computed with the plugin RMSDTT of VMD [42] and averaged over each system. The root-mean-square fluctuation (RMSF) was obtained with the Timeline plugin of VMD [42], for the residues 2–249 of the channel in each replica and averaged over each system. The hydrogen bonds and salt bridges between K245 and protein residues were analyzed in VMD [42] using the default parameters. The carbon atoms from the lipid membrane that entered the fenestrations and the pore of the channel during the simulations were counted and averaged over the whole trajectory for each system.

According to the RMSD average of each system, equilibrium was reached approximately after half of the production simulation. Consequently, the analyses were performed in the last 50 ns.

One snapshot every 5 ns per replica was saved to analyze whether the orientation of K245 residue was steered by (de)protonation or the fenestration state in the studied systems. The collected frames were employed as input for the conformer cluster program of the Maestro suite [43]. In order to identify the main differences between the cluster of K245′s side-chain conformers, we evaluated the minimal number of them (two clusters) per fenestration with the average linkage method.

The pK_a_ values of K245 were evaluated with PropKa v3.1 software [44,45], and system structures were collected every 0.5 ns to perform the pK_a_ calculation. Both the protein and the lipid membrane were explicitly considered for the calculation.

The radius of the lateral fenestrations during the MD simulations for the four studied systems was resolved with the HOLE program [26] in order to compare whether the presence/absence of the charge in K245 residue induced a change in the dimensions of the fenestrations. The same protocol employed for the TASK-2 homology models was used here.

The electrostatic potential (Φ) was carried out by solving the Poisson–Boltzmann equation using APBS v.1.4 software [46,47] during MD simulations of T2Tre1CC-K245^+^ and T2Tre2OO-K245^+^. The TASK-2 channel and the lipid membrane were collected every 1 ns and used explicitly in the calculation. The conduction pore of the structures was aligned in the *Z*-axis starting at the extracellular ion pathway (*Z* = −15) and finishing at the polar head of lipids located in the intracellular region (*Z* = 30), with *X* = *Y* = 0. The calculation consisted of measuring the Φ along the *Z*-axis every 0.25 Å for each frame with K245 protonated and turning off the charge. Both values were subtracted as shown in the formula from Figure 6, where ∆Φ represents the electrostatic potential of TASK-2 along the conduction pore with K245 protonated minus the electrostatic potential of TASK-2 when the charge of K245 residue was turned off. The aqueous solvent and ions were treated implicitly with a dielectric constant of ε = 80 and a probe radius of water molecules of 1.4 Å to define the molecular surface. The dielectric constant for the TASK-2 channel and the lipid membrane was set to ε = 2.

### 4.3. Heterologous TASK-2 Expression and Electrophysiological Measurements (Patch Clamp)

HEK-293 cells cultures were grown in Dulbecco’s Modified Eagle Medium/Nutrient Mixture F-12 (DMEM-F12) media containing 10% fetal bovine serum with 1% penicillin/streptomycin. The DNA was transfected into HEK-293 cells with Xfect polymer (TakaraBio distributed by Gene X-Press, Santiago, Metropolitan Region, Chile) and a DNA ratio of 3:1 (plasmid encoding TASK-2 channel, GenBank accession no. AF319542, 0.75 µg–plasmid encoding for green fluorescent protein (GFP) as marker, 0.25 µg). The experiments were done one day after transfection at room temperature. Whole-cell patch clamp recordings were carried out using an amplifier (PC-501A, Warner Instruments) and borosilicate pipettes as described elsewhere [48,49]. Recording pipettes were filled with an intracellular solution containing (in mM) 145 KCl, 5 EGTA, 2 MgCl_2_, and 10 HEPES, adjusted to pH 7.4 with KOH. The recording solution contained (in mM) 135 NaCl, 5 KCl, 1 MgCl_2_, 1 CaCl_2_, 10 HEPES, and 10 sucrose, adjusted to pH 7.4 with NaOH. Ion channel currents were measured with a voltage protocol (400-ms steps from −100 mV to 100 mV with an increment of 10 mV and a holding potential of −80 mV). The TASK-2 blockade was analyzed with a +80 mV test pulse.

A norfluoxetine racemic mixture was acquired from Cayman Chemical Company (distributed by Genytec, Santiago, Metropolitan Region, Chile) and diluted in the recording solution to give the desired final concentrations, prior to the experimentation. To calculate norfluoxetine IC_50_ in the TASK-2 channel, the concentrations used were (in µM) 0.1, 1, 10, 50, 100, and 300.

The mutants TASK2-K245A and TASK2-K245V were done by PCR as described previously [50,51].

### 4.4. Molecular Docking

Using the T2Tre2OO homology model with K245 residues deprotonated, a double docking experiment was generated, one per open fenestration. The norfluoxetine (NFX) structures (*R* and *S*) were exported from the co-crystalized TREK-2 channel (PDB: 4XDK) with NFX [14], and its protonation states were assigned with the PropKa [44,45] tool from the Maestro suite [43], where both stereoisomers could be protonated on the nitrogen atom. These four NFX combinations (*R*-protonated, *R*-neutral, *S*-protonated, *S*-neutral) were prepared with Ligprep [52]. The grids were positioned in open fenestrations using as the grid center the crystallographic NFX site, previously aligning the structures of the T2Tre2OO model and the crystal 4XDK. The docking algorithm used was the Standard Precision (SP) of Glide [53]. Ten docking poses per ligand (40 in total) were obtained and ranked by docking energy. The best pose per subunit was chosen by comparing its orientation with NFX in the TREK-2 channel.

### 4.5. Free Energy Calculations

To calculate the energetic cost for the translocation of a K^+^ ion from the solvent to the central cavity of the TASK-2 channel in different configurations (T2Tre1CC-K245^0^, T2Tre1CC-K245^+^, T2Tre2OO-K245^0^, T2Tre2OO-K245^+^), the extended adaptive biasing force algorithm [54] (eABF) was used. The COLVAR module [55] implemented in NAMD [38,39] was used to define the collective variables.

The models were aligned with the *Z*-axis parallel to the selectivity filter. Positional restraints of 1 kcal∙mol^−1^∙Å^−2^ were applied to the protein backbone to maintain the configuration of the TASK2 models during the simulations. A cylinder with a radius of 3 Å was defined along the reaction coordinate to restrict the movement of the ion within this region, using upper and lower wall constants of 50 kcal∙mol^−1^∙Å^−2^. The reaction coordinate along the *Z*-direction was defined as the distance between the center of mass of the alpha carbon of the lower residues of the selectivity filter (T98 and T203) and a potassium ion located at 35 Å toward the cytosol (Figure 7A). Then, the reaction coordinate of 30 Å in length (from the *Z*-coordinates 40–10 Å) was divided into 12 non-overlapping windows of 2.5 Å/window and 0.1 Å/bin. The lower wall constants and upper wall constants were set to 20 kcal∙mol^−1^∙Å^−2^ for each window to guarantee the sampling at each point of the reaction coordinate. The extended fluctuation parameter (σ) was set to 0.1, and the full sample parameter was set to 1000 at each window. For each window, 25 ns of simulation time was run in triplicate, and the standard error of the mean was calculated. The total simulation time was 0.9 μs per system.

## 5. Conclusions

In conclusion, the structural basis of the intracellular pH sensing mechanism of the TASK-2 K_2_P channel that was elucidated here might contribute in the future to the rational design of molecules to interact with K245 and modulate TASK-2 channel function.

## Figures and Tables

**Table 1:** The pKa prediction of K245 residue over MD trajectories. The pKa prediction was computed over the last 50 ns of simulation and averaged over three replicas (*n* = 300) for each system: T2Tre2OO-K245^+^, T2Tre2OO-K245^0^, T2Tre1CC-K245^+^, and T2Tre1CC-K245^0^. The calculation was carried out by considering only the channel and membrane atoms explicitly. K245 total corresponds to the average ± SD of the system protonated and neutral (*n* = 1200). The pK_1/2_ value reported by Niemeyer et al. [7] is used as a parameter to compare the pKa of K245 predicted in K245 total for open (T2Tre2OO model) and closed (T2Tre1CC model) states of the fenestrations.

TASK-2 Simulations						
		T2Tre2OO		T2Tre1CC		
System	Subunit	Average	SD	Average	SD	pK_1/2_ Experimental
K245^+^	A	8.65	0.63	7.95	0.43	~8.0
K245^+^	B	8.98	0.38	7.75	0.40	
K245^0^	A	7.27	0.37	6.86	0.35	
K245^0^	B	6.88	0.29	6.79	0.25	
K245 Total	All	7.93	0.98	7.34	0.63	

## Supplementary Materials

Supplementary materials including Table S1 and Figures S1–S4 can be found at https://www.mdpi.com/1422-0067/21/2/532/s1.
