# Supplementary material for: Elucidating the Structural Basis of the Intracellular pH Sensing Mechanism of TASK-2 K2P Channels
Source: Int J Mol Sci. 2020 Jan 14;21(2):532. doi: 10.3390/ijms21020532 (PMC7013731; doi:10.3390/ijms21020532)
Supplement: Supplementary file 1 [file ijms-21-00532-s001.pdf]

Supplemental Information: Elucidating the structural basis of the intracellular pH sensing mechanism of TASK-2 K_2P channel

	PDB ID	Fenestration state	CAP domain	Ligand presence	Mutant	Ion Occupancy	Resolution	References
TWIK-1	3ukm	O-O	n-DS	---	---	S0-S1-S2-S3-S4	3.4	Miller et al., 2012
TRAAK	3um7	O-O	n-DS	---	---	S0-S1-S2-S3-S4	3.3	Brohawn et al., 2012
	4i9w	C-O	DS	---	---	S0-S1-S2-S3-S4	2.8	Brohawn et al., 2013
	4wfe	C-C	DS	---	---	S0-S1-S2-S3-S4-SCav	2.5	Brohawn et al., 2014
	4wff	C-O	DS	lipid	---	S0-S1-S2-S3-S4	2.5	Brohawn et al., 2014
	4wfg	C-C	DS	---	---	S0-S1-S2-S3-S4-SCav	3.0	Brohawn et al., 2014
	4wfh	O-O	DS	lipid	---	S0-S1-S2-S3-S4	3.0	Brohawn et al., 2014
	4rue	O-C	DS	---	G124I	S0-S1-S2-S3-S4-SCav	3.3	Lolicato et al., 2014
	4ruf	O-C	DS	---	W262S	S0-S1-S2-S3-S4-SCav	3.4	Lolicato et al., 2014
TREK-1	4twk	C-C	DS	---	---	S2-S3-S4	2.6	Dong et al., 2014.
	6cq8	C-C	DS	lipid & ML335	---	S0-S1-S2-S3-S4-SCav	3.0	Lolicato et al., 2017
	6cq9	C-C	DS	lipid & ML402	---	S1-S2-S3-S4-SCav	2.8	Lolicato et al., 2017
	6cq6	C-C	DS	lipid	---	S0-S1-S2-S3-S4-SCav	3.1	Lolicato et al., 2017
TREK-2	4bw5	C-C	DS	---	---	S1-S2-S3-S4	3.2	Dong et al., 2015
	4xdj	O-O	DS	---	---	S2-S3-S4	3.9	Dong et al., 2015
	4xdk	O-O	DS	NFX	---	S2-S3-S4	3.4	Dong et al., 2015
	4xdl	O-O	DS	Br-FIUOx	---	S2-S3-S4	3.6	Dong et al., 2015
TASK-1	6rv2	O-O	DS	---	---	S1-S2-S3-S4	3.0	Rödström et al.
	6rv3	O-O	DS	BAY 1000493	---	S1-S2-S3-S4	2.9	Rödström et al.
	6rv4	O-O	DS	BAY 2341237	---	S1-S2-S3-S4	3.1	Rödström et al.

Table 1: Crystallographic information of K_2P members. Legend: O = open fenestration; C = closed fenestration; n-DS = non-Domain Swapped; DS = Domain Swapped; S_0 = K^+ coordination site above to the selectivity filter (SF). S_1-S_4 = K^+ coordination sites within the SF. S_{Cav} = K^+ coordination site in the inner cavity. NFX = norfluoxetine; Br-FIUOx = brominated fluoxetine derivative.

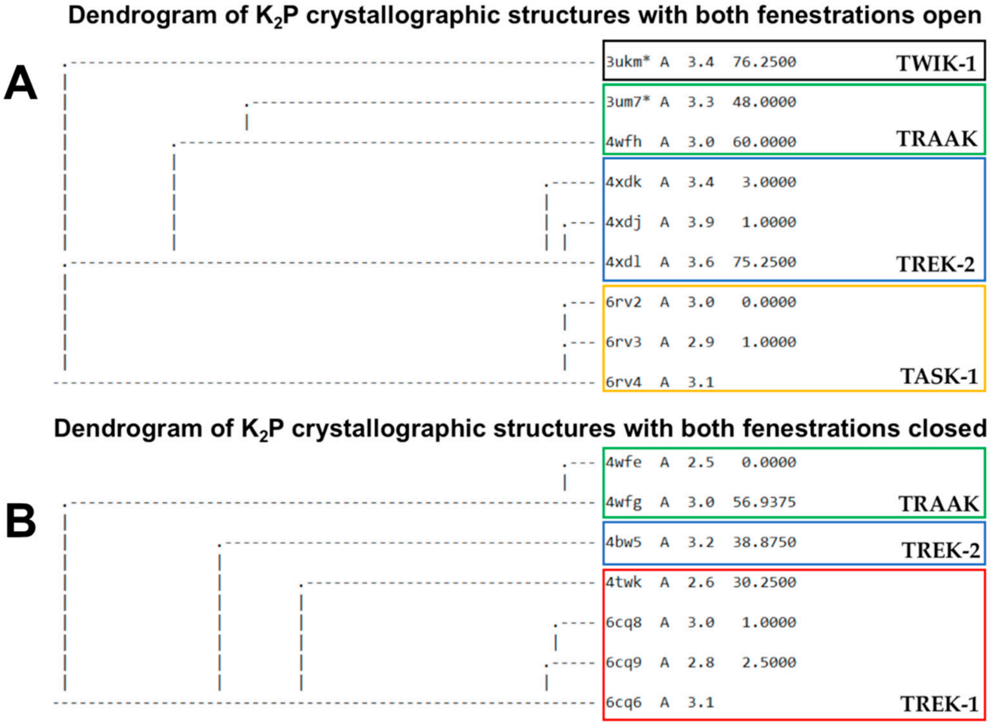

Figure 1: Dendrograms of K2P crystallographic structures. The clustering tree represents the sequence and structural comparison of K2P crystallographic data belonging to the sets with both fenestrations open A) and closed B). Only the dendrograms of the chain A of each crystallographic structures are depicted because the clustering for both chains is very similar. The information on the right side of the tree shows the PDB ID (e.g. 3ukm), chain or subunit (in this case only chain A), resolution of the crystal structure (e.g. 3.4 Å), a measure of the differences regarding to the root of the branch (e.g. 3ukm is 76.25 units far from the root TREK-2 4xdl and TASK-1 6rv4), and finally the name of the channel. As greater the distance as more different the structures.'*' in 3ukm and 3um7 represent a non-domain-swapped conformation.

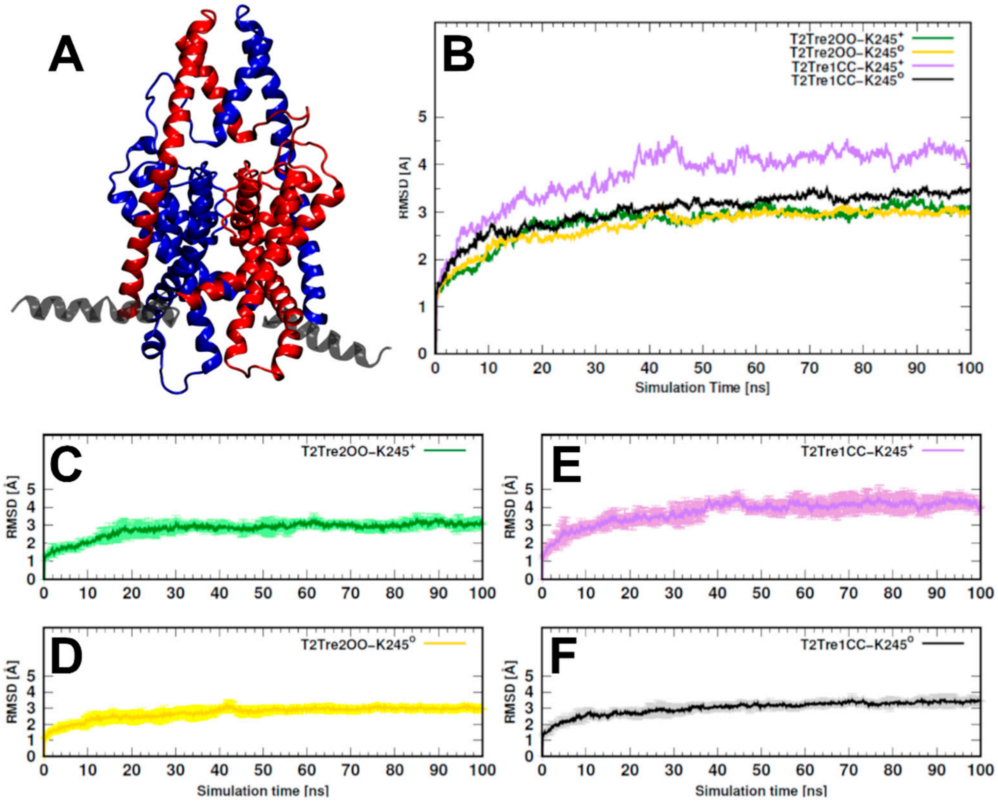

Figure 2: RMSD values for TASK-2 homology models during MDs. The time dependence of the RMSD values calculated over the residues 2 to 249 for both monomers. A) An example of TASK-2 based in T2Tre1CC showing both monomers in red and bluecolor from the residue 2 to 249, the transparent gray region correspond to the residues 250 to 278 (C-terminal) was not considered for the RMSD calculation. B) RMSD averaged over 3 replicas for the four systems studied. Average and standard deviation of RMSD values for T2Tre2OO-K245^{+} in green color depicted in C) and T2Tre2OO-K245^{0} in yellow color shown in D). Correspondingly to C) and D) but for T2Tre1CC model are shown in E) and F).

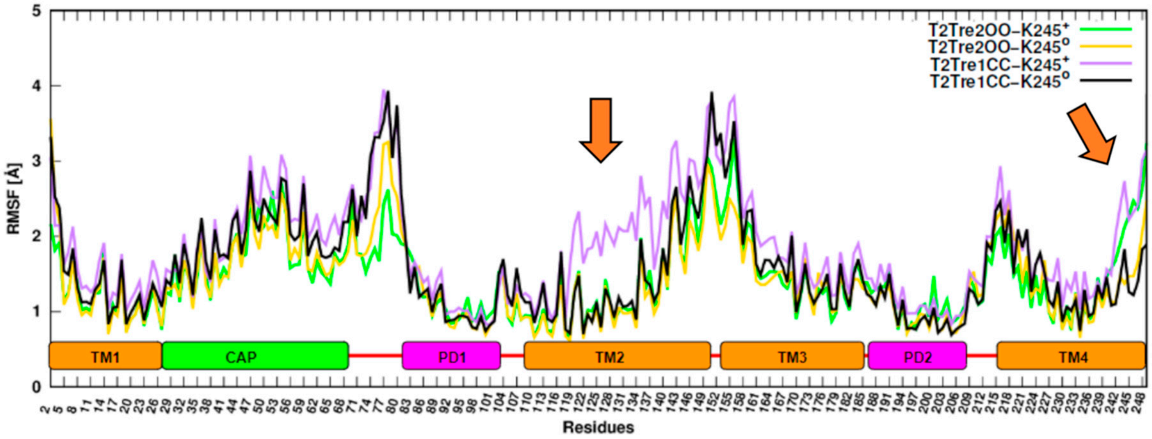

Figure 3: Root-mean square fluctuation (RMSF) plot during the simulation time (100 ns). RMSF values were averaged over 3 replicas and both chains for each studied system: T2Tre2OO-K245^{+} and T2Tre2OO-K245^{0} are in green and yellow lines, respectively. T2Tre1CC-K245^{+} and T2Tre1CC-K245^{0} are in purple and black lines, respectively. The orange boxes below to the RMSF plot represent the position of the four transmembrane helices of TASK-2. The CAP structure position is denoted by a green box, and the first and second pore domains (PD1 and PD2) are shown as purple boxes. The red line indicates the loops in TASK-2 channel. The orange arrows represent the biggest fluctuations in TM2 and TM4 helix.

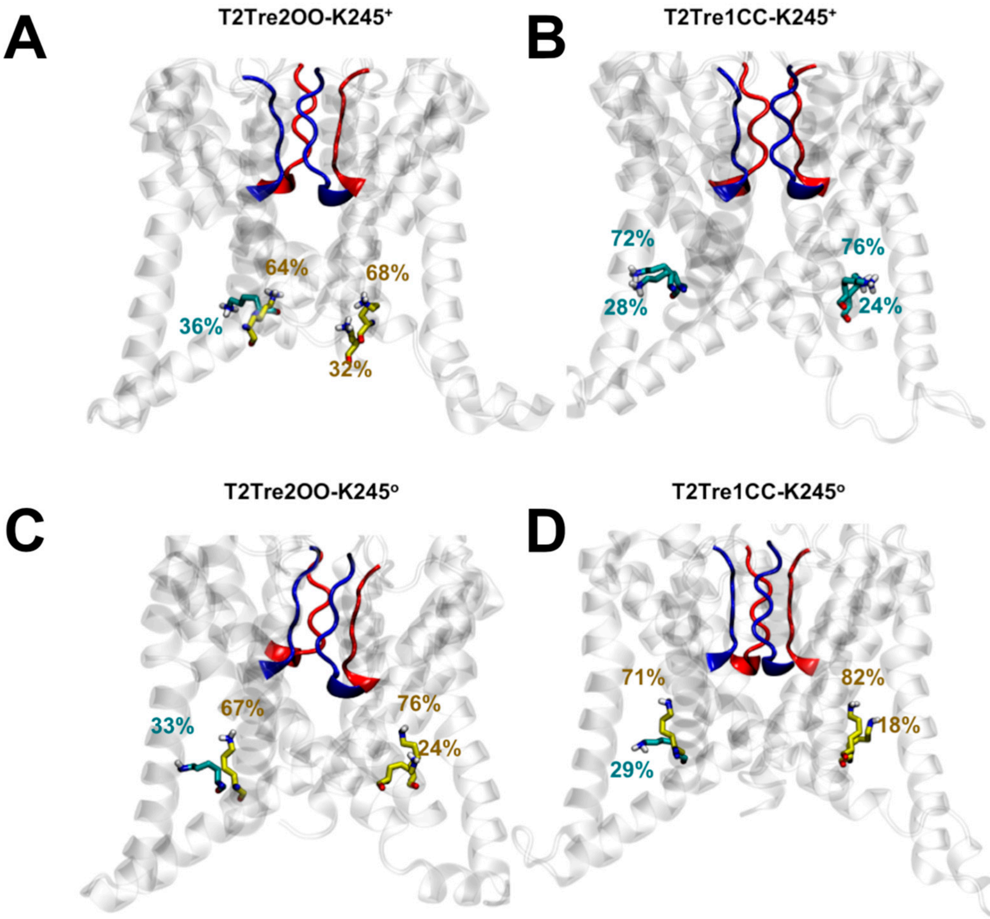

Figure 4: Clustering of K245 rotamers. During the last 50 ns of the simulation (50 frames *per* replica) was analyzed the side-chain position of K245 residue classifying its orientation into two clusters per subunit. The percentage number in yellow and cyan colors are the clusters with the biggest and lowest number of elements (K245 residues) based in their similarity, respectively. The residues K245 depicted in licorice are median element representing its cluster for A) T2Tre2OO-K245$^+$, B) T2Tre1CC-K245$^+$, C) T2Tre2OO-K245^0, and D) T2Tre1CC-K245^0 in each monomer. The cartoon representation in red and blue colors shows the selectivity filter of TASK-2 channel.

Video 1: **MD simulation of the TASK-2 homology model (T2Tre2OO) with K245 neutral and K245 protonated.** TASK-2 homology model with K245 neutral (yellow, left) and protonated (green, right) during 100 ns of MD simulations. Note the lack of TM4 movement toward TM2 in the presence of K245$^+$(right).
